# The Effects of Dementia Knowledge on Dementia Worry, Attitudes, Social Comfort, and Affect

**DOI:** 10.1093/geroni/igaa057.916

**Published:** 2020-12-16

**Authors:** Alexandria Ebert, Maya Huggins, Hannah Wagner, Zachary Lightcap, Tessla Muir, Mariah Faulkenberry, Amy Fiske

**Affiliations:** West Virginia University, Morgantown, West Virginia, United States

## Abstract

Previous work has demonstrated that personhood-based knowledge of dementia is associated with less fear of dementia and greater social comfort with persons with dementia. Nonetheless, a causal link has not been tested. We examined the effects of types of dementia knowledge on dementia worry, fear, and social comfort, as well as affect more broadly and attitudes toward dementia, which have not previously been examined. Participants (N = 338) aged 19-78 (M = 44.82, SD = 16.66) were randomized into one of five experimental conditions: biomedical-knowledge (BK; read biological and medical facts about dementia); personhood-based knowledge (PBK; read accounts written by persons living with dementia); both BK and PBK; baseline control; and active control. Participants then completed outcome measures. Significant effects of knowledge on dementia worry (p < .05) as well as personal distress, empathic concern, and negative affect (ps < .001) emerged; groups did not significantly differ in attitudes, comfort, or dementia fear. Specifically, participants in the BK and PBK conditions exhibited significantly higher levels of personal distress than those in the control conditions. Similarly, participants in the PBK condition had significantly higher levels of negative affect than those in the control conditions and significantly higher levels of dementia worry than those in the baseline control condition. Participants in the PBK condition also had significantly higher levels of empathic concern than those in the biomedical knowledge and control conditions. Results suggest that although reading about dementia induces negative affect, it also induces empathic concern.

